# Aberrant *GSTP1* promoter methylation is associated with increased risk and advanced stage of breast cancer: a meta-analysis of 19 case-control studies

**DOI:** 10.1186/s12885-015-1926-1

**Published:** 2015-11-19

**Authors:** Cheng Fang, Xue-Mei Wei, Xian-Tao Zeng, Fu-Bing Wang, Hong Weng, Xinghua Long

**Affiliations:** 1 Department of Laboratory Medicine, Center for Gene Diagnosis, Center for Evidence-Based and Translational Medicine, Zhongnan Hospital of Wuhan University, Wuhan, 430071 P.R. China; 2 Department of Nursing, Affiliated Hospital of North Sichuan Medical College, Nanchong, 637000 P.R. China

**Keywords:** Glutathione S-transferase P1, GSTP1, Breast cancer, Promoter methylation, Meta-analysis

## Abstract

**Background:**

Glutathione S-transferase P1 (*GSTP1*) has been reported to function as a tumor suppressor gene in various types of human cancers. Aberrant methylation of tumor-related genes at the promoter regions can inactivate genes, which is important in the carcinogenesis of breast cancer. However, the role of *GSTP1* promoter methylation in the occurrence of breast cancer and its relationship with tumor stage and histological grade has not been fully elucidated. Thus, we carried out a meta-analysis to yield a more accurate association.

**Methods:**

A systematically literature search was made on PubMed, EMBASE and Web of Science databases for eligible studies. The odds ratio (OR) and 95 % confidence interval (95 % CI) were calculated by RevMan 5.2 software. Subgroup and sensitivity analyses were conducted to explore the source of heterogeneity.

**Results:**

Eventually, 17 articles involving 19 case–control studies were included in the present meta-analysis. Overall, the pooled results indicated that aberrant *GSTP1* promoter methylation was significantly associated with the risk of breast cancer (OR = 7.85, 95 % CI = 5.12–12.01; Caucasians OR = 7.23, 95 % CI = 3.76–13.90 and Asians OR = 11.71, 95 % CI = 5.69–24.07). Furthermore, our results revealed that *GSTP1* promoter methylation was more often observed in late-stage breast cancer patients compared with early-stage ones (OR = 1.84, 95 % CI = 1.32–2.58). However, no significant association was identified between *GSTP1* promoter methylation and histological grade (OR = 0.74, 95 % CI = 0.43–1.26).

**Conclusions:**

The results indicated that *GSTP1* promoter methylation probably plays an important role in breast carcinogenesis, which could serve as an effective biomarker for the diagnosis and monitor of breast cancer.

**Electronic supplementary material:**

The online version of this article (doi:10.1186/s12885-015-1926-1) contains supplementary material, which is available to authorized users.

## Background

Breast cancer, a heterogeneous disease, is by far the most common malignancy that affects females. It has been reported that an estimated 1.7 million new cases of breast cancer were diagnosed with nearly 522,000 related deaths worldwide in 2012 [1]. Moreover, incidence rates differ between regions with a lifetime risk of 1 in 3 women in Asia and 1 in 8 women in the United States [2]. Despite intensive research, the molecular mechanism of cancer development is still not fully understood. Generally, the interplay between genetic and environmental risk factors has played an important role in the etiology of breast cancer [3]. In recent years, increasing evidence has shown that epigenetic changes of tumor-related genes are involved in the pathogenesis and development of breast cancer, and could be used as indicators of cancer diagnosis and treatment [4–6].

Glutathione-S-transferases (GSTs) are a family of enzymes involved in the detoxification of carcinogenic and cytotoxic substances by catalyzing their conjugation with reduced glutathione [7, 8]. Among the isoenzymes, the pi-class GST (GSTπ) encoded by the *GSTP1* gene is implicated in a large variety of detoxification and metabolism reactions, which prevent cells from genome damage and cancer initiation [9, 10]. The *GSTP1* gene is a tumor suppressor gene and locus on chromosome 11q13 [11]. Aberrant methylation of the *GSTP1* often occurs in different cancer types including those of liver, prostate, and breast cancer [12, 13]. Moreover, the silencing of *GSTP1* gene expression induced by promoter methylation has been found to be implicated in the pathogenesis of breast cancer [14].

To date, several studies have investigated the methylation patterns of the *GSTP1* in breast cancer patients, yet the data are greatly variable due to differences among studies. Therefore, we conducted a meta-analysis of the published clinical studies to evaluate the effect of *GSTP1* promoter methylation on breast cancer patients.

## Methods

### Eligible criteria

Eligible studies included in this meta-analysis should meet the following standards: (1) assessed the association between *GSTP1* promoter methylation and breast cancer; (2) independent case–control studies; (3) all patients met the clear diagnostic criteria for breast cancer; (4) provided sufficient data about the methylation levels of *GSTP1* in tissue or blood samples of cancer patients and normal controls; (5) the methylated *GSTP1* was detected by polymerase chain reaction (PCR) based methylation assays.

### Literature search

This meta-analysis was reported according to the checklist of the Meta-analysis of Observational Studies in Epidemiology (MOOSE) guidelines (Additional file 1: Table S1). We systematically searched related clinical studies regarding the association between *GSTP1* promoter methylation and breast cancer via PubMed, EMBASE and Web of Science databases (up to January 31, 2015). The key terms: (breast OR mammary) And (cancer OR neoplasm OR tumor OR carcinoma) And (GSTP1 OR glutathione S-transferase P1) And (methylation OR hypermethylation) were used. The references cited in the selected studies were also scanned for relevant studies.

### Data extraction

Data extraction was conducted independently by two reviewers from the included studies. The recorded information for each study contained the following: First author’s name, year of publication, patients’ ethnicity, sample type, sample size, tumor stage, histological grade, *GSTP1* methylation frequencies and the methylation detection methods. All selected studies used normal samples as controls, which were composed of normal breast tissues from breast cancer patients and normal samples from non-cancer people. Of these studies, we combined stage 0, I and II as early-stage, stage III and IV as late-stage, which were defined by AJCC staging system [15]. As for histological grade, Grade I and II were defined as low-grade, Grade III was defined as high-grade [16].

### Statistical analysis

Odds ratios (ORs) and their 95 % confidence intervals (CIs) were used to evaluate the association. Heterogeneity was quantified by the Cochran *Q* test with statistical significance set at *P* < 0.10 and *I*^2^ statistics. If there was no statistical heterogeneity among studies (*P* ≥ 0.10 and *I*^2^ < 40 %), we used the fixed-effect model to pool the results; otherwise, the random-effects model was applied [17]. Moreover, subgroup analyses, which were stratified according to the patients’ ethnicity, sample type and detection methods were performed to explore potential sources of heterogeneity and the differences among them. In the presence of heterogeneity, sensitivity analysis was conducted by omitting a single study in each turn to see whether a particular omission could influence the overall estimate. The funnel plots were applied to assess publication bias if the included number of studies was no less than nine. All above analyses were carried out using the Review Manager 5.2 software (Cochrane Collaboration, Oxford, UK). In addition, the effect of possible publication bias was evaluated using the Egger’s test [18] and trim-and-fill method [19] by STATA 12.0 software.

## Results

### Studies selection and characteristics

**Fig. 1:**
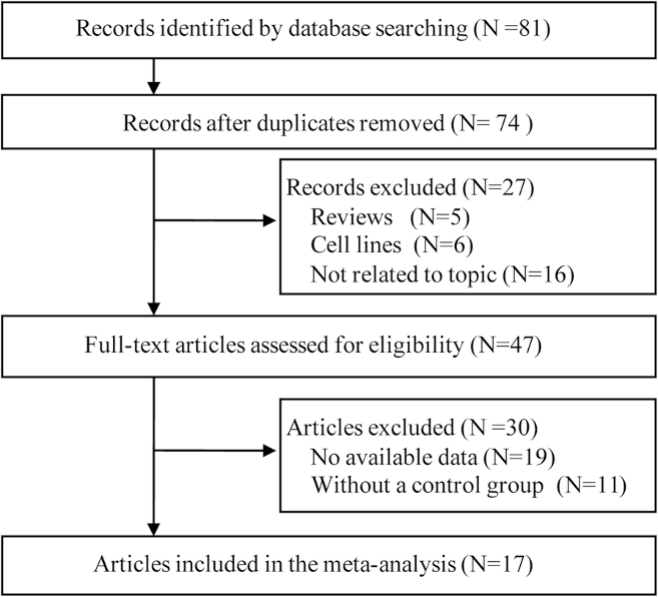
The flow diagram of the study selection process

After being selected in accordance with our inclusion criteria, 17 articles involving 19 case–control studies [20–36], comprising 1,647 cases and 559 controls were finally included, the publication years of the selected studies ranged from 2003–2014. Figure 1 showed the process of study selection.

Ten studies were conducted among Caucasians [20, 21, 24–26, 28, 30, 32, 35, 36], seven studies among Asians [23, 29, 31, 33, 34], one study among Africa [22] and the other one was among mixed populations [27]. Tumor tissues and blood samples were used to detect the methylation status of *GSTP1* promoter. Moreover, the methylated levels of *GSTP1* were assessed using a variety of PCR based methylation assays composing of methylation-specific PCR (MSP), quantitative MSP (QMSP), pyrosequencing, MethyLight assay, and methylation-specific multiplex ligation-dependent probe amplification (MS-MLPA). Characteristics of all included studies were summarized in Table 1.

**Table 1:** Main characteristics of included studies

Study ID	Ethnicity	Sample type	Case/ control	Methods (techniques)	GSTP1 (M/N)		Stage (M/N)		Grade (M/N)	
					Cacer	Normal	Early-stage	Late-stage	Low-grade	High-grade
Jeronimo 2003 [20]	Caucasian	Tissue	27/12	MSP (Non-quantitative)	17/27	0/12	-	-	-	-
Shinozaki 2005 [21]	Caucasian	Tissue	151/10	MSP (Non-quantitative)	32/151	0/10	-	-	-	-
Hoque 2006 [22]	African	Blood	90/38	QMSP (Quantitative)	12/47	0/38	6/24	22/66	-	-
Lee 2007 [23]	Asian	Tissue	85/15	MSP^+^ (Non-quantitative)	32/85	0/15	-	-	-	-
Pasquali 2007 [24]	Caucasian	Tissue	15/15	Pyrosequencing (Quantitative)	9/15	2/15	-	-	-	-
Jeronimo 2008 [25]	Caucasian	Tissue	66/12	QMSP (Quantitative)	33/66	2/12	-	-	-	-
Hoque 2009 [26]	Caucasian	Tissue	112/32	QMSP (Quantitative)	22/112	3/32	-	-	-	-
Brooks 2010 [27]	Mixed	Blood	50/99	QMSP (Quantitative)	2/50	7/99	-	-	-	-
Matuschek 2010 [28]	Caucasian	Blood	76/16	MethyLight assay (Quantitative)	14/76	1/16	3/39	9/31	-	-
Sharma 2010 [29]	Asian	Tissue	100/15	MSP (Non-quantitative)	25/100	1/15	8/51	17/49	15/48	8/28
		Blood	100/30		22/100	1/30	6/51	16/49	13/48	8/28
Moelans 2011 [30]	Caucasian	Tissue	72/9	MS–MLPA (Semi-quantitative)	32/72	0/9	-	-	4/25	4/14
Park 2011 [31]	Asian	Tissue	85/30	MethyLight assay (Quantitative)	25/85	0/30	-	-	6/30	8/20
Kornegoor 2012 [32]	Caucasian	Tissue	108/10	MS–MLPA (Semi-quantitative)	47/108	0/10	-	-	-	-
Yamamoto 2012 [33]	Asian	Tissue	94/53	MSP^+^ (Non-quantitative)	45/94	1/53	26/56	19/38	-	-
		Blood	159/87		21/159	2/87	3/68	9/57	-	-
Jung 2013 [34]	Asian	Tissue	60/60	MS–MLPA (Semi-quantitative)	10/60	2/60	10/53	0/7	6/40	4/20
Klajic 2013 [35]	Caucasian	Tissue	219/6	Pyrosequencing (Quantitative)	142/219	0/6	53/85	74/108	-	-
de Groot 2014 [36]	Caucasian	Tissue	21/10	Gel-based MSP (Semi-quantitative)	9/21	0/10	-	-	-	-

*MSP* methylation-specific PCR, *QMSP* quantitative MSP, *MS-MLPA* methylation-specific multiplex ligation-dependent probe amplification, *MSP*^*+*^ based on MSP with slight modifications, *M* the number of methylations, *N* number of total

### Overall and subgroup analyses

**Fig. 2:**
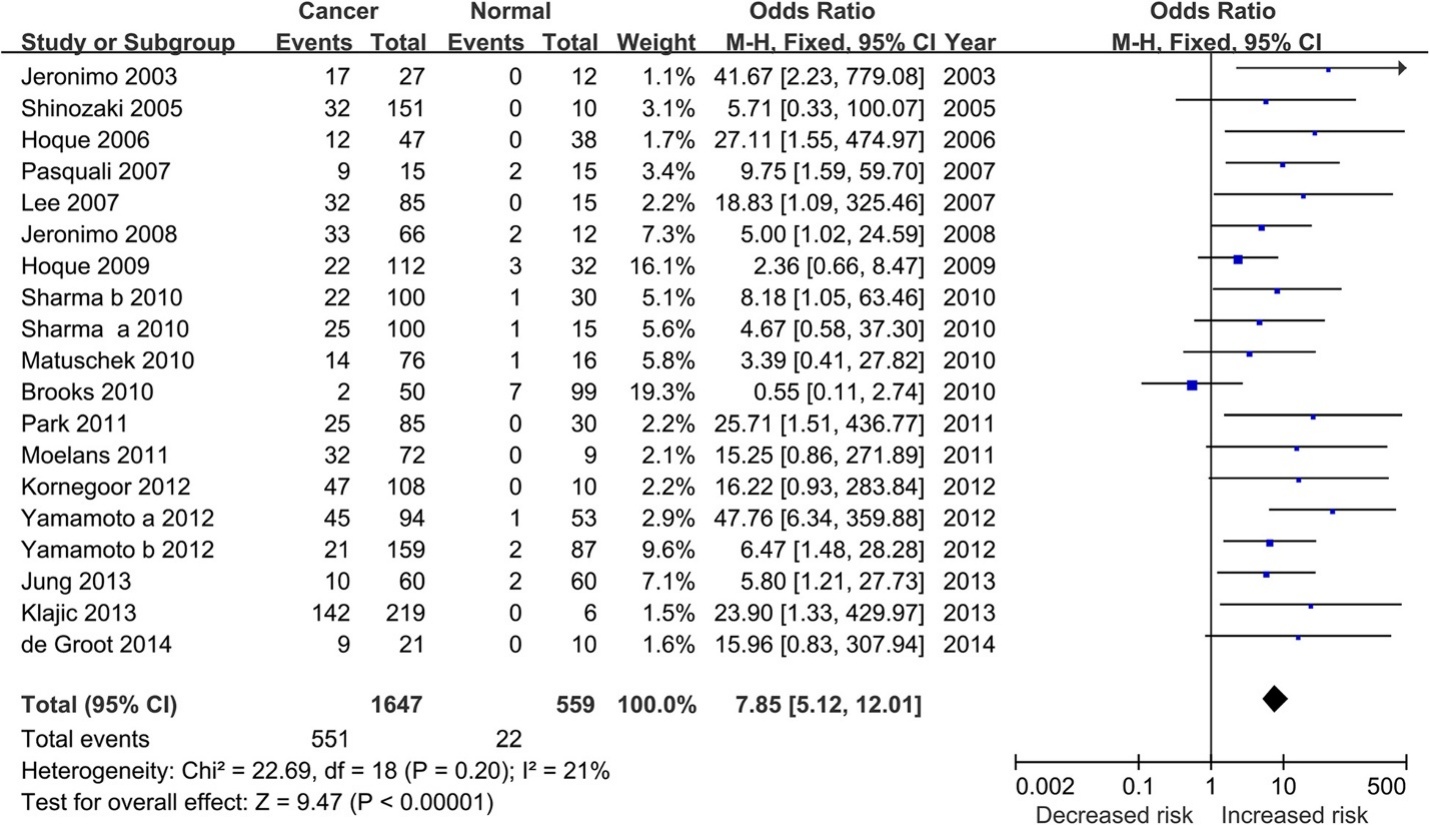
Forest plot of the association between *GSTP1* methylation and breast cancer risk based on a fixed-effect model. The squares and horizontal lines correspond to the OR and 95 % CI

Our results showed that breast cancer exhibited significantly higher frequency of *GSTP1* methylation than normal controls (OR = 7.85, 95 % CI = 5.12–12.01, Fig. 2). Moreover, subgroup analyses were performed to identify the influence of abnormal *GSTP1* promoter methylation on the risk of breast cancer. Ethnicity-stratified analysis revealed that there were statistical associations between *GSTP1* promoter methylation and increased breast cancer risk among both Caucasians (OR = 7.23, 95 % CI = 3.76–13.90) and Asians (OR = 11.71, 95 % CI = 5.69–24.07). After stratified by sample type, we found that aberrant methylation of *GSTP1* was correlated with the risk of breast cancer detected in tissue (OR = 10.32, 95 % CI = 5.97–17.85) as well as blood samples (OR = 4.02, 95 % CI = 1.12–14.38). After stratified by method, significant associations between *GSTP1* promoter methylation and the risk of breast cancer were observed in all of the subgroups (Quantitative: OR = 4.73, 95 % CI = 1.84–12.12; Semi-quantitative: OR = 10.33, 95 % CI = 3.32–32.10; Non-quantitative: OR = 12.55, 95 % CI = 5.72–27.55). Above results could be reviewed in Table 2.

**Table 2:** Overall and subgroups analyses of *GSTP1* methylation and breast cancer risk

Study groups	Number	OR (95 % CI)	Heterogeneity		
			P*h*	I^2^ (%)	χ^2^
Overall	19	7.85 (5.12, 12.01)	0.20	21	22.69
Ethnicity					
Caucasians	10	7.23 (3.76, 13.90)	0.67	0	6.65
Asians	7	11.71 (5.69, 24.07)	0.61	0	4.53
Sample type					
Tissue	14	10.32 (5.97, 17.85)	0.58	0	11.39
Blood	5	4.02 (1.12, 14.38)	0.07	54	8.64
Method					
Quantitative	8	4.73 (1.84, 12.12)	0.08	45	12.84
Semi-quantitative	4	10.33 (3.32, 32.10)	0.86	0	0.77
Non-quantitative	7	12.55 (5.72, 27.55)	0.61	0	4.51

*N* number of trials, *OR* odds ratio

**Fig. 3:**
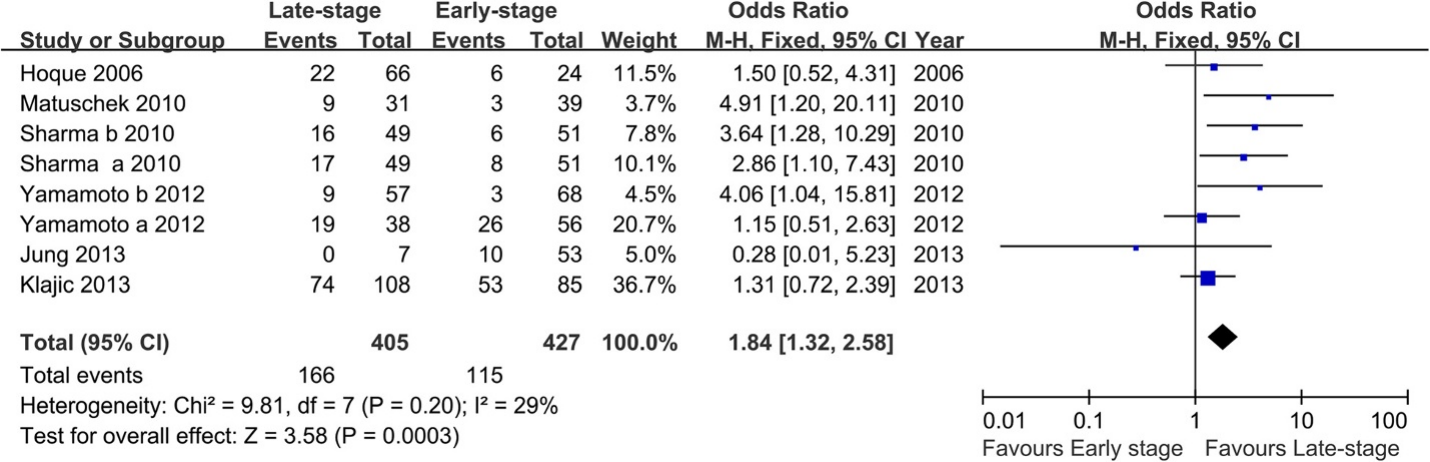
Forest plot of the association between *GSTP1* methylation and tumor stage based on a fixed-effect model. OR and 95 % CI were calculated

In addition, eight studies [22, 28, 29, 33–35] comprising 832 patients were pooled for the OR in evaluating the association between *GSTP1* methylation and tumor stage. The results revealed that aberrant *GSTP1* methylation was more often observed in late-stage patients compared with early-stage ones (OR = 1.84, 95 % CI = 1.32–2.58, Fig. 3). However, the pooled OR of five studies showed that there was no significant association between *GSTP1* methylation and histological grade (OR = 0.74, 95 % CI = 0.43–1.26) (Table 3).

**Table 3:** Association of *GSTP1* methylation and tumor stage/histological grade in breast cancer

Study groups	Number	OR (95 % CI)	Heterogeneity		
			P*h*	I^2^ (%)	*χ* ^2^
Stage	8	1.84 (1.32, 2.58)	0.20	29	9.81
Grade	5	0.74 (0.43, 1.26)	0.69	0	2.27

*N* number of trials, *OR* odds ratio

### Sensitivity analysis

The results showed that moderate heterogeneity existed in investigating the correlation of *GSTP1* methylation and breast cancer risk detected in blood samples and quantitative method by subgroup analysis (Table 2). Then, a sensitive analysis was used to find the heterogeneous study. After removal of the study by Brooks et al. [27], the heterogeneity presented in blood samples was reduced from *I*^2^ = 54 % (*P*^*h*^ = 0.07) to *I*^2^ = 0 % (*P*^*h*^ = 0.71), the heterogeneity in quantitative method was also reduced from *I*^2^ = 45 % (*P*^*h*^ = 0.08) to *I*^2^ = 0 % (*P*^*h*^ = 0.43), suggesting it might be the heterogeneous study. However, the pooled ORs were not significantly changed in sensitivity analyses, in which each study was deleted at one time, suggested the stability of our results.

### Publication bias

**Fig. 4:**
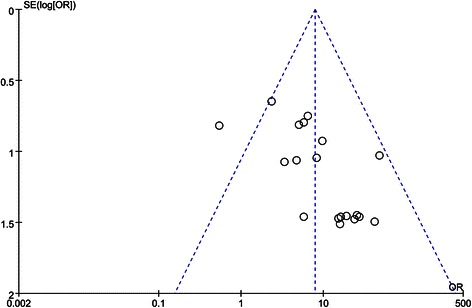
Funnel plot for evaluating publication bias test for *GSTP1* methylation and breast cancer risk. The standard error of log (OR) of each study was plotted against its log (OR)

**Fig. 5:**
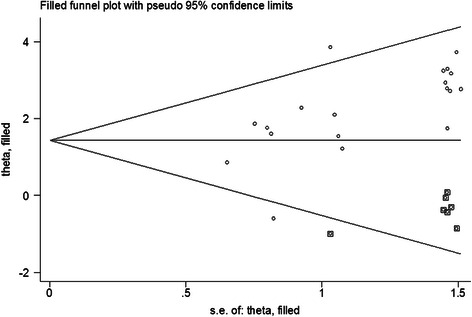
Funnel plot of publication bias test for *GSTP1* methylation and breast cancer risk after trim-and-fill method. Logor natural logarithm of OR, horizontal line mean effect size

Visual inspection of the funnel plot in Fig. 4 shows an asymmetry, which indicates the presence of publication bias in evaluating *GSTP1* methylation and breast cancer risk. Egger’s test also display statistical evidence of asymmetry (*P* = 0.003). Then, the trim-and-fill method was applied to adjust this bias and calculate the number of unpublished studies that could lead to asymmetry (Fig. 5). The estimated OR adjusted by trim-and-fill method was similar to the original estimate (OR = 4.20, 95 % CI = 2.75–6.41), indicating that our analyses were reliable and robust. For limited number of studies, the investigation of publication bias with tumor stage and histological grade were not examined.

## Discussion

GSTP1 is a member of the metabolic enzymes family, which has significant implications in the prevention of cancer initiation upon exposure to carcinogens [11, 13]. Absence of GSTP1 expression is found in approximately two thirds of the patients with breast cancer, suggesting it might play an important role in breast carcinogenesis [23]. It has also been demonstrated that the hypermethylation of *GSTP1* gene promoter frequently occurs in breast cancer and may result in inactivation of GSTP1 expression, thus lead to cancer progression [13].

The current meta-analysis demonstrated that the methylation level of *GSTP1* was significantly higher in breast cancer patients than that in normal controls, which indicated its potential role in the etiology of breast cancer. This was in accordance with the results of previous studies [22–24]. We also performed subgroup analyses to further explore the potential effects of the patients’ ethnicity, sample type and detection method on the association of *GSTP1* promoter methylation with the risk of breast cancer. The results revealed that *GSTP1* promoter methylation was closely associated with the risk of breast cancer in both Caucasians (OR = 7.23, 95 % CI = 3.76–13.90) and Asians (OR = 11.71, 95 % CI = 5.69–24.07), whereas, the correlation was stronger in Asians than in Caucasians. The reasons might include differences in genetic backgrounds, environments and sample size. After stratified by sample type, we found that aberrant methylation of *GSTP1* was correlated with the risk of breast cancer detected in tissue (OR = 10.32, 95 % CI = 5.97–17.85) as well as blood samples (OR = 4.02, 95 % CI = 1.12–14.38). Moreover, a high concordance between tumor and blood DNA methylation of *GSTP1* was reported in studies conducted on paired tumor tissue and blood samples from breast cancer patients [29, 33]. Yamamoto et al. compared the gene methylation status in serum DNA before and after surgery in patients with primary breast cancer, and demonstrated that the origin of blood methylated DNA was the tumor tissue because patients with aberrant *GSTP1* methylation in serum DNA collected before surgery were found to be negative for gene methylation after surgery [33]. This indicated that blood DNA methylation of *GSTP1* could reflect alterations in the tumor and the ease of obtaining blood samples makes it a potential biomarker for diagnosis of breast cancer. In the present meta-analysis, the small number of patients, various ethnicity groups and different time of sample collection may contribute to relatively extended confidence intervals. To date, a diversity of PCR based methylation assays were developed and widely used to measure methylation in clinical specimens, classified as quantitative, semi-quantitative and non-quantitative techniques [37]. Several papers have compared MS–MLPA (semi-quantitative) with pyrosequencing (quantitative) or MSP (non-quantitative) and showed a good concordance between MS–MLPA and pyrosequencing [30, 37]. Since different methylation assays were applied to detect the methylation levels of *GSTP1* in the studies included in this meta-analysis, we also performed subgroup analysis based on methods to explore potential sources of heterogeneity and the differences among them. As a result, significant associations were identified as detected by quantitative, semi-quantitative and non-quantitative techniques, suggested these methods have the same effect in *GSTP1* methylation detection. However, when only quantitative analyses of *GSTP1* promoter methylation in blood DNA are pooled, no significant association was observed (data not shown). It is hypothesized that the small sample size may lead to false-negative results. Furthermore, different patient materials and the choice of different primer sets between different studies may influence the results. Aberrantly methylated genes are frequently found in human cancers but rarely in normal controls, and their presence is not an exclusive attribute of metastatic cancer. Examination of body fluid from patients with early stage or organ-confined tumors may also reveal positive results [28]. Our study showed that the methylation level of *GSTP1* increased significantly in late-stage compared to the early stage breast carcinomas, suggested that breast cancer patients with *GSTP1* promoter hypermethylation may have a biologically aggressive phenotype.

Breast cancer is a complex multifactorial disease that is driven by genetic and epigenetic alterations, which cause aberrant gene function [38]. Previous study declared that the genetic variation of *GSTP1* affected its enzymatic activity and detoxifcation ability, thereby contributing to breast cancer susceptibility [10]. Epigenetic alterations including DNA methylation and histone modifications which occur in transformed cells are identified as an early event during tumor development [35, 39]. In breast cancer, hypermethylation of promoter CpG islands has been described as the main epigenetic pathway to inactivate genes involved in various aspects of cellular function [30]. It has been reported that *GSTP1* is capable of inhibiting tumor growth by its interaction with the c-Jun N-terminal kinase (JNK1) signaling [11], suggesting its role as a tumor suppressor gene. Additionally, because of its detoxifying effects on the anticancer agents, *GSTP1* may also affect the sensitivity of breast tumors to chemotherapy, emerged as a novel therapeutic target [7, 9].

To our knowledge, this is the first meta-analysis comprehensively performed to assess the relationship between *GSTP1* promoter methylation and the incidence of breast cancer. Nevertheless, a number of potential limitations should be acknowledged. First, the effects of potential risk factors such as age, subtype and hormone receptor status on the current results of this meta-analysis could not be eliminated for lack of detailed information. Second, meta-analysis is a secondary analysis and the heterogeneity is the major issue in genetic studies [40–43]. Of course, our meta-analysis also suffered this issue and we performed subgroup analyses to explore the origin. The results showed that different methylation methods, source of controls and cut-offs positivity of hypermethylation might contribute to heterogeneity. Third, only published clinical studies were selected in this meta-analysis, some unpublished and negative studies may contribute to publication bias. Since studies with statistically positive results were easier to publish than those with negative results, publication bias is inevitable. However, the estimated OR adjusted for publication bias by trim-and-fill method was not substantially changed. Fourth, although our initial search has no language restrictions, only articles published in English and Chinese finally were reviewed. This due to the language ability and the right to use databases of our team, and also might result in some bias.

## Conclusions

In conclusion, our meta-analysis suggested a strong association between *GSTP1* promoter methylation and breast cancer risk. Thus, aberrant *GSTP1* promoter methylation could be a helpful biomarker for the early screening of breast cancer. However, given the limitations elaborated above, high quality studies with larger sample sizes should be employed in further research.

## Additional file

Additional file 1: Table S1. MOOSE checklist in current meta-analysis. (DOC 73 kb)

## Abbreviations

AJCC: American Joint Committee on Cancer
CI: Confidence interval
GSTP1: glutathione s-transferase P1
MOOSE: Meta-analysis of Observational Studies in Epidemiology
MS-MLPA: methylation-specific multiplex ligation-dependent probe amplification
MSP: methylation-specific polymerase chain reaction
OR: odds ratio
QMSP: quantitative methylation-specific polymerase chain reaction
RevMan: Review Manager
